# Physiological basis of interactive responses to temperature and salinity in coastal marine invertebrate: Implications for responses to warming

**DOI:** 10.1002/ece3.7552

**Published:** 2021-05-01

**Authors:** Gabriela Torres, Guy Charmantier, David Wilcockson, Steffen Harzsch, Luis Giménez

**Affiliations:** ^1^ Alfred‐Wegener‐Institut Helmholtz‐Zentrum für Polar‐ und Meeresforschung Biologische Anstalt Helgoland Helgoland Germany; ^2^ CNRS Ifremer IRD UM Marbec Université Montpellier Montpellier France; ^3^ Institute of Biological, Environmental and Rural Sciences Aberystwyth University Aberystwyth UK; ^4^ Department of Cytology and Evolutionary Biology Zoological Institute and Museum University of Greifswald Greifswald Germany; ^5^ School of Ocean Sciences College of Environmental Sciences and Engineering Bangor University Menai Bridge UK

**Keywords:** *Carcinus maenas*, climate change, coastal zone, larva, mRNA expression, multiple stressors, osmoregulation, salinity, temperature

## Abstract

Developing physiological mechanistic models to predict species’ responses to climate‐driven environmental variables remains a key endeavor in ecology. Such approaches are challenging, because they require linking physiological processes with fitness and contraction or expansion in species’ distributions. We explore those links for coastal marine species, occurring in regions of freshwater influence (ROFIs) and exposed to changes in temperature and salinity. First, we evaluated the effect of temperature on hemolymph osmolality and on the expression of genes relevant for osmoregulation in larvae of the shore crab *Carcinus maenas*. We then discuss and develop a hypothetical model linking osmoregulation, fitness, and species expansion/contraction toward or away from ROFIs. In *C. maenas*, high temperature led to a threefold increase in the capacity to osmoregulate in the first and last larval stages (i.e., those more likely to experience low salinities). This result matched the known pattern of survival for larval stages where the negative effect of low salinity on survival is mitigated at high temperatures (abbreviated as TMLS). Because gene expression levels did not change at low salinity nor at high temperatures, we hypothesize that the increase in osmoregulatory capacity (OC) at high temperature should involve post‐translational processes. Further analysis of data suggested that TMLS occurs in *C. maenas* larvae due to the combination of increased osmoregulation (a physiological mechanism) and a reduced developmental period (a phenological mechanisms) when exposed to high temperatures. Based on information from the literature, we propose a model for *C. maenas* and other coastal species showing the contribution of osmoregulation and phenological mechanisms toward changes in range distribution under coastal warming. In species where the OC increases with temperature (e.g., *C. maenas* larvae), osmoregulation should contribute toward expansion if temperature increases; by contrast in those species where osmoregulation is weaker at high temperature, the contribution should be toward range contraction.

## Graphical abstract

Exposure to increased temperature leads to an increased capacity to osmoregulate in larvae of *Carcinus maenas*. Acclimation to diluted seawater improves osmoregulatory capacity at low salinities.

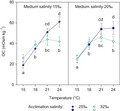

## 1 INTRODUCTION

Climate change is driving multiple modifications in environmental conditions (e.g., temperature, humidity) of both terrestrial and aquatic organisms (Boyd et al., 2018; Gattuso & Hanssen, 2009; Gunderson et al., 2016; Helmuth et al., 2010). Environmental change is multivariate, and therefore, organisms are exposed to the simultaneous action of several environmental drivers (Sokolova et al., 2012, Torres et al., 2019; Torres et al., 2020; previously referred as stressors: Crain et al., 2008; Folt et al., 1999; Piggott et al., 2015). Thus, environmental drivers can operate on biological systems (e.g., organisms) in an interactive mode and responses (e.g., survival) cannot be accurately predicted by evaluating the action of each driver in isolation (Boyd et al., 2018; Côté et al., 2016; Crain et al., 2008; Gunderson et al., 2016; Kroeker et al., 2013; Piggott et al., 2015). Interactive responses are classified in two general categories: synergistic or antagonistic. Relevant examples to climate change are, for instance, synergistic effects of increased temperature combined with food limitation or elevated CO_2_ (i.e., the combined effect of both stressors is larger than the sum of each separate effect: Giebelhausen & Lampert, 2001; Przeslawski et al., 2015; Schiffer et al., 2014; Torres & Giménez, 2020). By contrast, increased temperature and reduced salinity can operate antagonistically: That is, warming appears to mitigate the negative effects of low salinity (or other drivers) on the performance and fitness in some coastal marine organisms (Lange & Marshall, 2017; Spitzner et al., 2019; Torres et al., 2020).

Currently, there is a gap in our knowledge about the mechanisms that underlie organismal responses to multiple environmental drivers. The capacity to better understand and predict effects of climate change through multiple drivers requires knowledge of the mechanisms driving such effects (De Laender, 2018; Galic et al., 2018; Orr et al., 2020; Thompson et al., 2018). In particular, responses occurring at the individual, population, or species levels are based on physiological mechanisms driving tolerance, in addition to biotic interactions (Ames et al., 2020; Baert et al., 2016; Pörtner, 2010; Sokolova et al., 2012; Somero, 2010, 2011). Hence, understanding physiological responses to multiple environmental drivers is the starting point toward better models of biotic responses to climate change.

Coastal organisms, occurring in estuaries or regions of freshwater influence (ROFIs: Simpson, 1997), are frequently exposed to low salinity, which usually reduces organismal performance (Anger, 2003; Kinne, 1971). ROFIs are coastal areas influenced by freshwater runoff (associated with estuaries) and are widespread along the world coastal zones (e.g., North European Seas, East coast of the Americas, SE Asia, and West coast of Africa). ROFIs are nursery areas for many economically important species. Predictions for changes in salinity at ROFIs vary regionally (IPCC 2013) and seasonally (Robins et al., 2016) but all share the same fate in one regard: Temperature is expected to increase over the century. Thus, at the ROFIs, warming will create a new environment within which organisms face additional stressors such as reduced salinity. Hence, a key question is how such organisms will respond to reductions of salinity in the context of increased temperatures. At present, there is no testable mechanistic model predicting species’ responses under such scenarios. More in general, reviews on the combined effect of multiple drivers on performance and fitness bring different results about whether responses to temperature and salinity are antagonistic or synergistic (Crain et al., 2008; Przeslawski et al., 2015).

The aim of this paper is twofold: First, we explore the mechanistic basis of an antagonistic effect of temperature and salinity on a model species. Second, we use our results and information from the literature to propose a testable hypothetical model linking osmoregulation, interactive effects of temperature and salinity on organismal performance, and future changes in species distributions across coastal‐estuarine gradients in response to warming. We first focus on the antagonistic effect called thermal mitigation of low salinity stress (TMLS: Spitzner et al., 2019) which is common in coastal‐estuarine organisms (Anger, 1991; González‐Ortegón & Giménez, 2014; Janas & Spicer, 2008; Kinne, 1971). TMLS is defined as the mitigation of the negative effects of exposure to low salinity on performance or fitness (e.g., survival) by increased temperatures. The critical point is that in species exhibiting TMLS, moderate warming may mitigate the negative effect of low salinity on survival. We studied the mechanisms driving TMLS in the larval stages of the shore crab *Carcinus maenas*. The shore crab *C. maenas* is native to northern Europe and NW Africa but is a successful invader worldwide (Compton et al., 2010; Leignel et al., 2014; deRivera et al., 2007; Roman & Palumbi, 2004; Young & Elliot, 2020). *Carcinus maenas* is also representative of a large number of marine organisms that develop through a pelagic larval phase (Anger, 2006; Spitzner et al., 2018).

We focus on larval stages of *C. maenas* because we know that TMLS occurs in our study population (Spitzner et al., 2019), and the target stages are osmoregulators (Cieluch et al., 2004); yet, the effect of temperature on osmoregulation has never been studied in larvae of *C. maenas*. We are interested in larvae because they are more sensitive to environmental variation compared to the juvenile–adult stages (Pandori & Sorte, 2019), and variation in survival controls recruitment (Cowen & Spounagle, 2009; Giménez, 2004; Palumbi, 2003). For *C. maenas*, the first and last larval stages (zoea I and megalopa), as well as juveniles and adults, hyper‐osmoregulate at low salinity (Cieluch et al., 2004; Siebers et al., 1972); all these stages occur in coastal or estuarine waters. In contrast, advanced zoeal stages (zoea II‐IV) occur in open waters and do not hyper‐regulate at low salinities. Spitzner et al. (2019) found that increased temperatures (21–24°C) experienced during the early larval stages can mitigate the effect of low salinity (20‰) on larval survival and developmental time, as compared to larvae reared at temperatures experienced by the local population (<20°C; North Sea, German Bight, Wiltshire et al., 2010). Within a population, the strength of TMLS can vary due to parental influences, but evidence for TMLS is found in populations of the Irish Sea (Nagaraj, 1993; Torres et al., 2020) and in the Pacific coast of N. America where it is an invader (Hines et al., 2004).

Several mechanisms may drive TMLS in *C. maenas* larvae (and other organisms). For instance, more individuals may reach a given larval stage (or survive to maturity in other species) because high temperatures reduce the time of exposure to a stressor; we define this as “phenological mechanism” as it is based on a change in the timing of events in the life cycle (Post, 2020). In addition, there should be adaptive physiological mechanisms explaining the response. Based on previous work on other species (Campbell & Jones, 1989; Charmantier‐Daures et al., 1988; Flügel, 1963; Janas & Spicer, 2008), we hypothesize that increased temperatures cause an enhancement in the capacity of individuals for extracellular osmoregulation. Extracellular osmoregulation (here referred to as “osmoregulation”) is defined as the active regulation of the concentration of osmotically active substances in the hemolymph or blood (Charmantier, 1998; Henry et al., 2012; Lignot & Charmantier, 2015; Lucu & Towle, 2003; McNamara & Faria, 2012; Péqueux, 1995; Rahi et al., 2018). Osmoregulation is an adaptive mechanism keeping organisms at optimal functioning, maintaining for instance the acid–base balance (Whiteley, 2011; Whiteley & Taylor, 2015), sustaining growth (Torres et al., 2011), and perhaps contributing to tolerance to ocean acidification (Whiteley et al., 2018). Osmoregulation is achieved through the active uptake of ions from the surrounding “diluted” water by transport cells (ionocytes), located in the epithelium of specialized organs (gills in adults; branchiostegites in larval stages of crustaceans: Cieluch et al., 2004, 2007). Ion uptake occurs through the concerted action of several proteins including the enzyme Na^+^‐K^+^‐ATPase (Cieluch et al., 2007; Ituarte et al., 2016; Lucu & Towle, 2003; Mackie et al., 2005; Thuet et al., 1988; Torres et al., 2007), and ion co‐transporters (e.g., Na^+^‐K^+^‐_2_Cl^‐^ symporter). The action of the above‐mentioned proteins may involve several processes including the up‐regulation of gene expression of those enzymes and co‐transporters (Faleiros et al., 2017; Ituarte et al., 2016; Luquet et al., 2005; Serrano & Henry, 2008; Xu & Liu, 2011).

We report on three experiments designed to determine the mechanisms driving the mitigation effect produced by elevated temperature in larvae of *C. maenas* exposed to moderately low salinities (TMLS). In the first and second experiments, we studied the effect of temperature and salinity on the osmoregulatory capacity (OC) of zoeae I and megalopae, respectively. In the third experiment, we evaluated the combined effects of salinity and temperature on the levels of mRNA expression of the genes coding for the Na^+^‐K^+^‐ATPase and the Na^+^‐K^+^‐_2_Cl^‐^ symporter in zoeae I. Then, we discuss and integrate our results into a wider framework and formulate a testable model showing the contribution of osmoregulation to range expansion and contraction of coastal‐estuarine species toward or away from ROFIs.

## 2 METHODS

### 2.1 Experimental design and procedures

Experiments were carried out with larvae hatched from berried females and megalopae collected from the field at the island of Helgoland (North Sea, German Bight) during the reproductive period in spring–summer (Experiments 1 and 2: OC; Experiment 3: gene expression patterns). During embryogenesis, berried females were kept in separated 2L aquaria in oxygenated and filtered (0.2 µm) natural seawater (32.5‰) in a temperature‐controlled room at 18°C with a 12:12 hr light:dark cycle. All experiments were run following standard methods of larval rearing (Spitzner et al., 2019; Torres & Giménez, 2020; Torres et al., 2011) using filtered natural seawater, constantly aerated. Larvae were reared in temperature‐controlled rooms (at 15, 18, 21, 24°C) and at two acclimation salinities (see Table S1 for experimental design). Salinity (expressed as salt content in “‰”) was manipulated by diluting natural seawater (32.5‰) with appropriate amounts of tap water and adjusting values using a salinometer (see Table S2 for details on acclimation and test salinities and the corresponding values of osmotic pressure expressed in mOsm/kg). Water was changed daily: Experimental glass bowls were rinsed and cleaned, larvae were fed with freshly hatched *Artemia* sp. nauplii, and dead individuals were removed from the cultures.

### 2.2 Experiment 1: Osmoregulation in zoeae I

We evaluated how temperature modified the OC in larvae reared under different acclimation salinities and subsequently exposed to different test salinities. OC is defined as the difference between the osmolalities of hemolymph and of the external medium at a given salinity under different conditions (Lignot et al., 2000). We therefore used a factorial design (see Table S1 for details) considering (a) acclimation salinities, that is, those experienced from hatching until initiation of the 24 h exposure to the test salinities, (b) test salinities, that is, those experienced during 24 h previous to the sampling of hemolymph, (c) temperature experienced from hatching until time of sampling of hemolymph. The acclimation salinities and temperatures were chosen based on a previous study showing TMLS (Spitzner et al., 2019). The test salinities were those where larvae are known to significantly hyper‐regulate while survival rates are high (>60%: Cieluch et al., 2004); larvae reared in seawater are near the osmotic equilibrium and thus OC ≈ 0 mOsm/kg.

To avoid effects associated with developmental processes related to the molt cycle, OC was quantified after exposure to the test salinities in zoeae I at intermolt (i.e., more than 50% of the molt cycle: period between hatching of zoea I and molt to zoea II occurred at the acclimation salinities). Freshly hatched larvae were first assigned to replicate groups of different acclimation salinities and temperatures (see Table S1), group‐reared in 500‐ml glass bowls until the time of exposure to the test salinities at a density: 0.1 individual × ml^−1^ (see Experiment 3 for rearing details). Because acclimation salinity and temperature affect developmental time (Table S3), we took special care to ensure that organisms were sampled at intermolt at the appropriate time (see Table S3). Zoeae I from each combination were assigned randomly to the two test salinities by placing individuals in petri dishes for 24 h. Previous experiments had shown that an exposure time of less than 24 h is sufficient for hemolymph osmolality to become stable in *C. maenas* larvae (Cieluch et al., 2004) and in other tested species (Charmantier, 1998). The osmotic pressure of the test salinities was expressed as osmolality (3.4‰ ≈ 100 mOsm/kg, 29.4 mOsm/kg ≈ 1‰, thus natural seawater at 966 ± 1 mOsm/kg = 32.5‰) which was determined with a micro‐osmometer (Model 3MO, Advanced Instruments) using 20 µl of each salinity (Table S2).

Larvae used to quantify hemolymph osmolality were quickly rinsed in deionized water, gently dried on a filter paper, and submersed in mineral oil to avoid evaporation and desiccation; the remaining water was then aspired using a micropipette. A second micropipette was inserted into the heart in order to extract the hemolymph (sample volume ~30 nl). Hemolymph osmolality was then determined with reference to the medium osmolality (i.e., test salinities) using nanoosmometry (Kalber‐Clifton nanoliter osmometer; Clifton Technical Physics), following Charmantier et al. (1998), Charmantier et al. (2002), and Cieluch et al. (2004). The results were expressed as OC, that is, the difference between the osmolality of the hemolymph and the medium (i.e., test salinities).

### 2.3 Experiment 2: Osmoregulation in megalopae

Osmoregulation in megalopae was quantified in larvae collected in the intertidal of Helgoland in summer. We opted for field collections because the large number of larvae needed for the experiments (80 megalopae =4 temperatures × 2 acclimation salinities × 1 test salinity × 10 megalopae) was difficult to obtain from cultures. Megalopae were taken from floating and benthic collectors deployed and collected daily, using standard techniques (Giménez et al., 2020). Collected megalopae were immediately transferred to the laboratory, assigned randomly to the different acclimation temperature and salinity treatments as in Experiment 1 (Table S1, Experiment 2), and reared following the same protocol as the zoeae I (see Experiment 1 for details) for 3 days, before exposure to the test salinities. At the appropriate time, megalopae (see Table S4 for n) were exposed to the test salinity for 24 h and then used for quantification of OC following the same techniques as those used for zoeae I (see Experiment 1). Due to low availability of megalopae, we only used one test salinity (20‰).

### 2.4 Experiment 3: Expression of genes related to osmotic stress in zoeae I

To assess the variation in the gene expression patterns caused by low salinities and elevated temperatures, we selected two target genes encoding for the ion‐transport enzyme Na^+^‐K^+^‐ATPase and the Na^+^‐K^+^‐2Cl^‐^ symporter (Towle & Weihrauch, 2001), and used the elongation factor 1 and ubiquitin‐conjugating enzyme E2 L3 as reference genes (Oliphant et al., 2018). The gene expression was evaluated in zoeae I from four separate females collected from the intertidal of Helgoland. Each replicate for the determination of the expression of mRNA consisted of 125 pooled zoeae I after exposure to the acclimation salinities until intermolt as in Experiment 1 (see Table S3 for details). In order to obtain the appropriate number of larvae for ca. 10 replicates (*n* = 10, as in the osmoregulation capacity measurements), the rearing of more than 10,000 larvae was required (*n* = 10 implies rearing more than 10 × 125 = 1,250 individuals per treatment; 1,250 × 8 = 10,000 larvae). Since we were not able to rear such a large number of larvae simultaneously, we used larvae harvested from four different females and took 3 replicates per female per treatment (all females together: *n* = 12 per treatment, 12,000 larvae were sampled). We are aware of the variation among females in the responses to temperature, salinity, and other stressors (Spitzner et al., 2019; Torres et al., 2020); therefore, we maintained the factor “Female” as a random factor in our statistical analyses and present the averaged values of all females as well as the data discriminated by female (see Results).

Upon hatching, larvae of each female were assigned at random to culture bowls for mass rearing. The rearing conditions were chosen in the range of temperatures and salinities where TMLS has been found (Spitzner et al., 2019), as for Experiment 1 (acclimation salinities: 25.0 and 32.5‰; temperatures: 15, 18, 21 and 24°C; see Tables S1 and S3 for details). Zoeae I were then sampled from the mass cultures at intermolt (i.e., >50% of the molt cycle occurred under the acclimation conditions) to avoid effects of the molt cycle (as for Experiment 1, see Table S3).

Three replicate samples (125 larvae each: see Tables S1 and S3 for details) were taken to determine the mRNA level of genes related to ion transport: Na^+^‐K^+^‐ATPase (*CamaNaK*) and Na^+^‐K^+^‐_2_Cl^‐^ symporter (*CamaCOT*) using elongation factor 1 (*CamaEL*) and ubiquitin‐conjugating enzyme E2 L3 (*CamaUB*) as reference genes (following Oliphant et al., 2018). In a PCR clean environment, larvae were quickly rinsed in distilled water, gently blotted dry with filter paper, placed in 1 ml of RNAlater^®^ to stabilize the RNA, and immediately frozen at −80°C for later analysis. In addition, three replicates (200 larvae each) of freshly hatched zoea from each female were collected to use as control samples.

Samples were quickly rinsed in DEPC water to eliminate the RNAlater^®^ and placed in PCR clean microcentrifuge tubes (kept at 4°C) containing TRIzol^®^ (Invitrogen). Total RNA was extracted from each replicate of pooled larvae by homogenizing the tissue directly in 500 μl TRIzol^®^ in a Qiagen TissueLyser LT (50 oscillations per minute). Extractions were carried out according to the manufacturer's instructions, except that the washing steps in 75% ethanol were repeated twice. Extracted RNA was resuspended in 20 μl *RNAse*‐free water before removal of contaminating gDNA with Turbo DNA‐free™ *DNAse* (Ambion) following manufacturer's instructions. Total RNA concentration (714 ± SE 18 ng/ml) was determined spectrophotometrically with a NanoDrop ND2000™ (Thermo Scientific). Three μl of RNA from each extraction was reverse‐transcribed using High Capacity™ cDNA synthesis kit reagents (Applied Biosciences) and random primers for 10 min at 25°C followed by 120 min at 37°C; the reaction was terminated by heating to 85°C for 5 min. The obtained cDNA was stored at −20°C until the quantitative PCR was performed.

Standard curves or the quantitative PCRs were developed for all genes (target: *CamaNaK* & *CamaCOT*, internal reference: *CamaUB* & *CamaEL*; sequences for all primers and probes in Table S5). Templates for standard curves were generated by reverse transcription of RNA extracted from transport tissues (gills) from adult *C. maenas* and appropriate dilution of the resulting cDNA. Two sets of multiplex TaqMan PCRs were done in 96‐well plates using the Bioline SensiFAST™ Probe Lo‐ROX kit following manufacturer's instructions. The qPCR mix (8 µl) consisted of 5 μl SensiFAST Probe Lo‐ROX mix, 0.4 μl of each primer (400 nM), 0.1 µl probe (100 nm), 1.2 µl DEPC water, and 2 μl cDNA (either standards for the calibration curves or unknowns). Selection of different reporter fluors allowed reactions to run in multiplex with the following combinations (NaK‐F & NaK‐R and COT‐F and COT‐R; UB‐F and UB‐R and EL‐F and EL‐R; see Table S5 for details). Quantitative PCRs (in duplicate plates as technical replicates) were run on an Applied Biosystems StepOne Plus™ machine using the following cycling parameters: 95°C for 2 min followed by 40 cycles of 5 s at 95°C and 30 s at 60°C. PCR amplification factor and efficiency were determined using the qPCR Efficiency Calculator Thermo Fischer Scientific (See Table S6 for details) with slopes calculated (GraphPad software) from the calibration curves performed with cDNA from gill tissues; standards were accepted only when amplification efficiency was >90%. Relative expression of target genes (*CamaNaK* and *CamaCOT*) was determined against the values from freshly hatched larvae as control using the 2^‐∆∆Ct^ method (Livak & Schmittgen, 2001; Pfaffl, 2006) modified to consider different amplification efficiencies and multiple reference genes (Hellemans et al., 2007; Vandesompele et al., 2002). All data were normalized to the geometric mean of the reference genes elongation factor 1‐alpha (*CamaEL*) and ubiquitin‐conjugating enzyme E2 L3 (*CamaUB*).

### 2.5 Statistical analyses

The OC data (Experiments 1 and 2) were analyzed through 3‐way factorial ANOVA (Underwood, 1997) with temperature, acclimation salinity, and test salinity as factors. Preliminary tests showed that residuals approached normal distribution and variances were homogeneous (Cochran test). For the mRNA expression data (Experiment 3), we used mixed modeling (Zuur et al., 2009) in order to control for the random variation associated with female of origin. In that case, analysis was based on generalized linear squares modeling using the *lme* function of the package *nlme* (Pinheiro et al., 2018), implemented in R (R Core Team, 2013). Thus, for the mRNA expression data, female of origin was considered a random factor in addition to the acclimation temperature and salinity used as fixed factors in a factorial design. Hypotheses for the mRNA expression data were evaluated through backward model selection; selection of the best random model was carried out through restricted maximum likelihood fitting while selection of the fixed terms was carried out through maximum likelihood. The best model was chosen using the adjusted Akaike information criterion (AICc). For each variable, the model showing the smallest AICc score was selected unless the lowest scored model was more complex than the next scored model and the corresponding difference between those models was ∆AICc <3; in that case, we tested the models using likelihood ratio tests. We also report *p*‐values associated with those models (see Table S7 for details).

## 3 RESULTS

### 3.1 Osmoregulation in zoeae I

**FIGURE 1:**
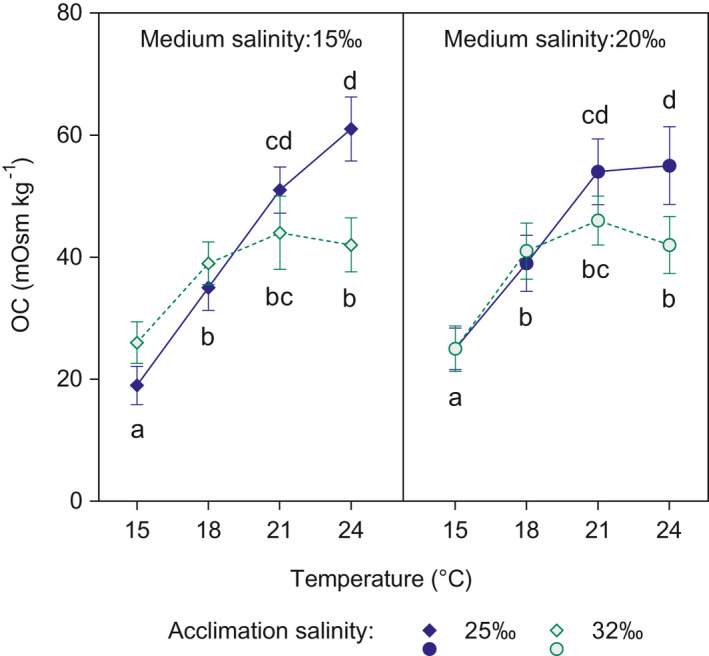
*Carcinus maenas*. Effect of temperature on the osmoregulatory capacity (OC) of zoeae I acclimated to 25.0‰ (blue symbols) and seawater (32.5‰, green symbols) during Experiment 1. OC was determined after exposure to the medium (test) salinities: 15.0 (diamonds in left panel) and 20.0‰ (circles in right panel). Values are shown as mean ± standard error (*n* = 10). Different letters show significant differences among treatments

All larvae, irrespective of the acclimation salinity or temperature, survived the exposure to the test salinities (=100% survival). The OC of zoea I larvae responded to the interactive effect of temperature and acclimation salinity (Figure 1 and Figure S1), and it was not significantly affected by the test (medium) salinity (15 or 20‰). There was a strong effect of temperature: The OC at the highest temperatures (21 and 24°C) was about two to three times higher than that shown by larvae kept at 15°C. Acclimation to low salinities enhanced the positive effect of high temperature on the OC. In larvae acclimated to seawater, the OC was significantly higher for those individuals reared in 18°C as compared to 15°C, but it reached a plateau in the range 18–24°C at ca. 40–45 mOsm/kg. By contrast, in larvae acclimated to 25‰, the OC increased with temperature up to a maximum of 57 mOsm/kg at 24°C.

### 3.2 Osmoregulation in megalopae

**FIGURE 2:**
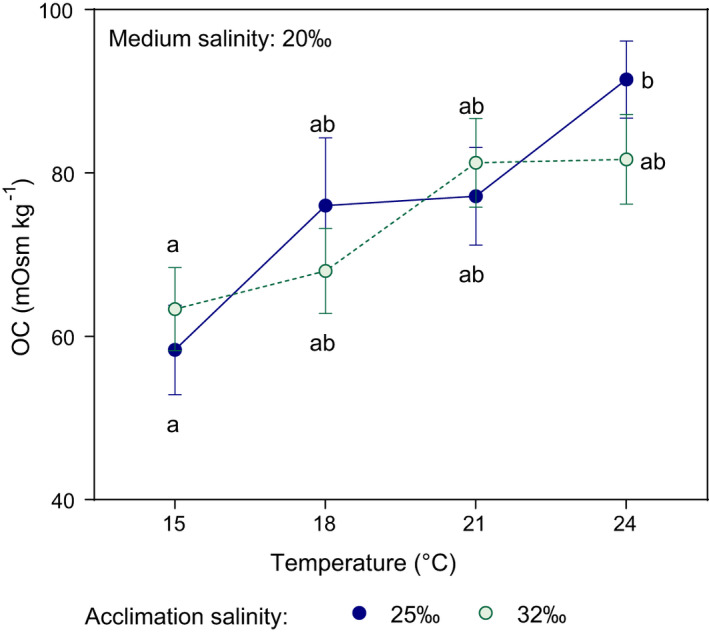
*Carcinus maenas*. Effect of temperature on osmoregulatory capacity of megalopae acclimated to 25.0‰ (blue circles) and seawater (32.5‰, green circles) during Experiment 2. OC was determined after exposure to the medium (test) salinity: 20.0‰. Values are shown as mean ± standard error (*n* = 5–8, see Table S4 for details). Different letters show significant differences among treatments

The OC of the megalopae increased with temperature, but was not significantly affected by the acclimation salinity (Figure 2). OC increased almost linearly with temperature, from ~60 mOsm/kg at 15°C to 85 mOsm/kg at 24°C; that is, temperature resulted in an increase of 40% in OC. By contrast, differences associated with acclimation salinity were not consistent across temperatures and resulted in a maximum of 12.5% increase from seawater to 25.0‰, occurring at 24°C.

### 3.3 Gene expression patterns in zoeae I

**FIGURE 3:**
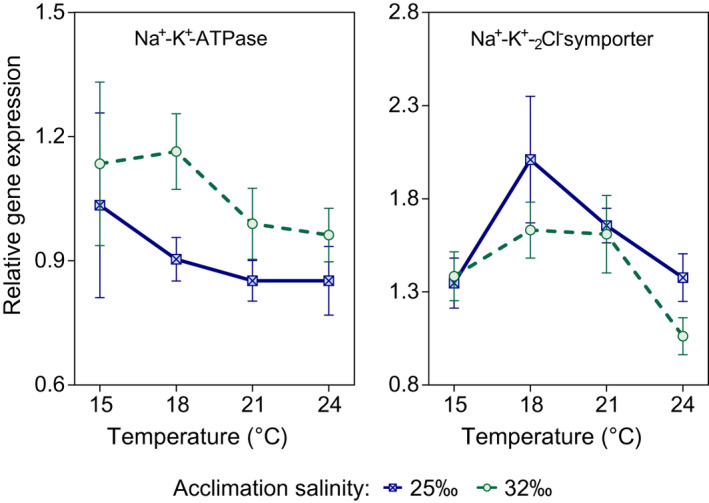
*Carcinus maenas*. Effects of acclimation salinity and temperature (Experiment 3) on relative expression of mRNA of Na^+^‐K^+^‐ATP (left panel) and Na^+^‐K^+^‐_2_Cl^‐^ symporter (right panel) in zoeae I. Data are shown as average values ±SE (*n* = 4 for all four females); acclimation to 25.0‰ is shown in blue and to natural seawater (32.5‰) in green. Data discriminated by female are shown in Figure S2

For the enzyme Na^+^‐K^+^‐ATPase (NaK), low acclimation salinity reduced the average gene expression by about 13% (Figure 3). Best models retained acclimation salinity (but not temperature) as the main driver of gene expression. There was also important variation in the response associated with the female of origin (Figure S2, left panel; Table S7). Plots of average gene expression by female did not show a consistent effect of temperatures although the prevailing pattern was a decreased gene expression at low salinity (Figure 3, left panel).

On average, gene expression of the Na^+^‐K^+^‐_2_Cl^‐^ symporter (COT) peaked at 18°C and showed a decrease toward lower or higher temperatures with a minimum at 24°C (Figure 3, right panel) where it achieved the largest decrease (ca. 26%). The best model retained temperature as the main driver of gene expression and also showed important variation in the response by female: Plots by female of origin showed that gene expression peaked at 18°C (fem 1, 3, 4) or 21°C (fem 2) but decreased expression was found consistently at 15 and 24°C (Figure S2, right panel).

## 4 DISCUSSION

We found that increased temperatures enhanced the capacity to osmoregulate in the zoea I and megalopa (Experiments 1 and 2). We also found (in zoeae I) subtle changes in expression of Na^+^‐K^+^‐ATP and Na^+^‐K^+^‐_2_Cl^‐^ symporter mRNA (NaK and COT, respectively) related to the acclimation salinity and temperature (Experiment 3). Because the results of the mRNA expression study were inconclusive, we still do not know which mechanisms operate at the cellular or molecular levels. However, the hemolymph osmolality appears to provide an integrative measure of the capacity to tolerate the combined effects of temperature and salinity. Information on osmoregulatory responses is important for the development of a predictive model for responses to warming and reduced salinity. However, such models require the establishment of links between physiology, performance, and species distribution (Ames et al., 2020). We divide the following discussion in four sections covering such links, for both the case of *C*. *maenas* and coastal‐estuarine species in general. We present a model showing the contribution of osmoregulation to fitness and species’ distribution in response to climate‐driven changes in temperature and salinity.

### 4.1 Thermally driven osmoregulation

The positive relationship between OC and increased temperature in both zoea I and megalopa of *C. maenas* matched previous observations in adult stages of coastal crustaceans (e.g., Campbell & Jones, 1989; Charmantier, 1975; Hagerman & Uglow, 1983; Janas & Spicer, 2008; Williams, 1960). The determinations of the OC (Experiments 1 and 2) showed an important and rapid response to salinity after exposure of larvae of *C. maenas* to the test salinities (15 and 20‰) for 24 h. In addition, measurements of OC in zoeae I showed that previous acclimation to low salinity (25‰) contributed to further increase the OC. Hence, it appears that osmoregulation results from responses to salinity at different time scales. We can ascribe the increase in osmoregulation to physiological plasticity because survival was >80% during the acclimation period (data not shown, see also Spitzner et al., 2019) and 100% during exposure to the test salinities (i.e., there was little opportunity for phenotypic selection). The two ‐ to threefold increase (ca. 20 mOsm/kg at 15°C to ca. 50–60 mOsm/kg at 24°C) in the OC found in *C. maenas* driven by temperature demonstrates a considerable amount of physiological plasticity. The increase is also important in the light of the existing information on interspecific variation in OC exhibited by crustacean larvae. Available data for decapod crustacean larvae show that the OC at comparable salinities (17‰) ranges between ~20 mOsm/kg, for coastal marine species (including *C*. *maenas*), and 140–200 mOsm/kg found in larval (zoea I and megalopa) stages of the Jamaican crab *Armases miersii*, developing in supratidal pools characterized by strong variations in salinity (Charmantier et al., 1998). The values of 60–90 mOsm/kg found in this study at 24°C (for zoeae I and megalopae, respectively) shifts *C*. *maenas* toward the mid‐range, characteristic of larvae of estuarine crabs which are released in (or return to) brackish waters (Charmantier et al., 2002; Cieluch et al., 2007). For example, the OC recorded for the first and last larval stages (zoeae I and megalopae) of the estuarine crab *Neohelice granulata* is 130–150 mOsm/kg at 17‰ (Charmantier et al., 2002). Thus, within the three patterns of ontogeny of osmoregulation described in crustaceans (Charmantier, 1998), variations in hemolymph osmolality values seem possible due to physiological plasticity; testing this hypothesis in a strongly hyperosmoregulating species such as *Armases miersii* would be worthwhile.

We evaluated, for zoeae I, if increments in relative mRNA expression for NaK and COT would contribute to the mechanism underpinning the thermally driven osmoregulatory plasticity (Experiment 3). As this is the first report on mRNA expression of NaK and COT in larvae of *C*. *maenas*, we hypothesized that the response would be similar to that found in adult gills (*C. maenas*: Jillette et al., 2011, Whiteley et al., 2018) and in other species (Havird et al., 2013; Serrano & Henry, 2008). We did not find evidence of such up‐regulation but instead mRNA levels were modestly down‐regulated at low salinity, for both NaK and COT. In addition, COT was slightly down‐regulated at the highest tested temperature. Down‐regulation occurred at the same salinity (25‰) where we found significant increase in OC (in Experiment 1: acclimation to 25‰ significantly increased the OC at high temperatures as compared to acclimation in seawater) and where Whiteley et al. (2018) found up‐regulation in adult *C. maenas*. We propose four explanations for these changes, although we think that only the last two explanations are likely to be correct. First, the increase in levels of mRNA expression that occur in osmoregulatory tissues may be masked when quantified using whole body samples. However, Lind et al. (2013) reported an increase in expression of variants of Na^+^‐K^+^‐ATPase in cyprids from the euryhaline barnacle *Amphibalanus improvisus* using samples of intact cyprids. Second, the time scale of the transcriptional response is much shorter than the time scale of exposure to low salinity in our experiment (3–5 days). If the transcriptional response in larvae is short, it is not consistent with that of adults, where up‐regulation was still observed after >3 months of exposure to low salinity (Whiteley et al., 2018); in addition, it is not consistent with studies showing that the transcriptional response can occur on a longer time scale (Faleiros et al., 2017, 2018). Third, the signal may be too weak because zoeae I of *C. maenas* are poor osmoregulators when compared to adults; at the tested low acclimation salinity (25‰), the OC of juvenile/adult crabs is >10 times higher than that of larvae (Cieluch et al., 2004: 749 mOsm/kg ~ 25‰ salinity). In addition, the tolerance to low salinity of *C. maenas* larvae is lower than that of *A. improvisus* (down to 5‰: Nasrolahi et al., 2012); *A. improvisus* may be a strong osmoregulator. Fourth, post‐translational adjustments (enzyme kinetic behavior: Corotto & Holliday, 1996) can occur more rapidly than transcriptional responses (Faleiros et al., 2017, 2018) and may explain the differences between OC and transcriptional responses. Additional adjustments may also occur through neurohormonal control (Lucu & Towle, 2003) and translocation of enzymes from intracellular vesicular stores to the membranes (McNamara & Torres, 1999).

### 4.2 Temperature, OC, and tolerance to low salinity

The positive effect of temperature on OC in zoeae I and megalopae of *C. maenas* (Figures 1 and 2) was consistent with the TMLS effect found previously (Spitzner et al., 2019): Larvae reared at the temperature range 21–24°C were more tolerant to low salinities than those reared at 15°C. There are two potential groups of mechanisms driving such responses. First, there is the effect of increased temperature that shortens the developmental time (“phenological mechanism”): If instantaneous mortality rates were constant, larval survival to a given stage (e.g., molting to zoea II) should increase with temperature simply because such larvae took less time to molt. Second, there is the effect generated by adaptive responses to low salinity such as osmoregulation; such responses should reduce the instantaneous mortality rate (here called “physiological mechanism”). Those mechanisms are not mutually exclusive, and they are likely operating simultaneously.

In order to check for evidence of a “physiological mechanism,” we used data reported by Spitzner et al. (2019, their Figure 3) to calculate the instantaneous mortality rates from hatching to molting to zoea II. These data were produced with larvae from the same local population as this study; it thus can be compared to the osmoregulatory patterns observed in Figure 1. Such calculations (Table S8) show that when TMLS occurred, the instantaneous mortality rates were two times lower in larvae reared at high temperature than those reared in low temperature. Hence, the “phenological mechanism” alone cannot explain TMLS, and instead, a “physiological mechanism” should also play a role. We argue in favor of osmoregulation as a potential “physiological mechanism,” because the capacity to osmoregulate provides organisms a steady state of body fluids, which contributes to the maintenance of performance and fitness (Charmantier, 1998; Charmantier et al., 2009; Péqueux, 1995; Whiteley, 2011).

An important question is whether there is evidence between changes in osmoregulation and TMLS. Our experiments do not provide a direct link because we had to measure osmoregulation (and hence kill the larvae) before the TMLS arises. Instead, we have indirect evidence in that TMLS occurs in the same population where we found the positive effect of temperature on OC. Additional evidence is provided by studies in temperate amphipods and shrimps (Hagermann & Uglow, 1983; Janas & Spicer, 2008; Kinne, 1952; Williams, 1960), showing decreased OC and survival under the combination of low salinity and low temperature. As stated by Kinne (1971), a disruption of active transport processes is expected below the low critical temperature.

More in general, evidence of a link between osmoregulation and tolerance to low salinity is widespread across adult crustaceans (Mantel & Farmer, 1983; Péqueux, 1995). Even small increases in OC, as 20 mOsm/kg, result in enhanced salinity tolerance, particularly in weak osmoregulators. For example, in the copepod *Eurytemora affinis*, slight increases in OC result in dramatic evolutionary shifts in adaptability to low salinity media (Lee et al., 2012). Evidence for crustacean larvae is given for instance by studies on stage‐dependent patterns of osmoregulation, showing that the increased OC is characteristic of species or stages that are able to survive over a wide range of salinities (Anger & Charmantier, 2000; Anger et al., 2008; Charmantier, 1998; Charmantier et al., 1998). Another piece of evidence is the positive relationship between OC and accumulation of reserves at low salinities existing in crustacean larvae (Torres et al., 2011). There is also evidence showing that the OC matches patterns of survival at the intraspecific level concerning the effect of salinity acclimation (Charmantier et al., 2002). Overall, we conclude that there is sufficient comparative evidence to propose a link between the effect of temperature on osmoregulation and on tolerance to low salinities.

### 4.3 Implications for climate change

If the effects of temperature on OC and phenology (timing of events such as molt or metamorphosis) are relevant to fitness, they may also drive changes in species’ distributions. From the standpoint of osmoregulation, responses should be species‐specific because osmoregulation can increase (Janas & Spicer, 2008; Kinne, 1971, this study), decrease (Burton, 1986; Weber & Spaargaren, 1970), or show little response to temperature (additional examples in Burton, 1986). From the standpoint of the phenological effects, survival should increase with temperature but such effect may vary across species depending on the slope of the development‐temperature curve. Phenological effects of high temperature (e.g., increased survival to metamorphosis) may counteract negative effects of temperature on osmoregulation (decreased survival) and may enhance positive effect of temperature on osmoregulation.

Concerning osmoregulation, there is evidence suggesting a link between OC and species distributions in crustaceans. For instance, Weber and Spaargaren (1970) noted differences between the effects of temperature on osmoregulation of species distributing toward cold versus warm latitudes: The shrimp *Crangon* s*eptemspinosa* shows a direct positive effect of temperature on osmoregulation (Haefner, 1969) and distributes further south than *C. crangon* with an inverse effect of temperature (Flügel, 1963). Weber and Spaargaren (1970) also discussed cases showing how thermally driven osmoregulation varied in “winter” and “summer” acclimated organisms in order to match the prevailing temperature conditions. Similar patterns have been found in more recent studies: For instance, Janas and Spicer (2008) found that osmoregulation at high salinities was impaired at low temperatures in large individuals of the shrimp *Palaemon elegans*, which perform winter migrations off the coast of Great Britain, but similar temperatures did not impair osmoregulation in smaller individuals, which remained in intertidal pools.

There is additional evidence (provided by various sources), suggesting a link between invasion of estuaries and the capacity to osmoregulate (Freire et al., 2008; Lee et al., 2012). Another source of evidence is the strong match between patterns of ontogenetic migration in marine crustaceans and concurrent ontogenetic changes in the capacity to osmoregulate (Anger & Charmantier, 2000, 2011; Anger et al., 2008; Charmantier et al., 1998, 2002; Cieluch et al., 2004, 2007). Those studies show that larval stages occupying or crossing water masses characterized by low (and varying) salinity (estuaries, rivers, and tidal pools) are osmoregulators while those occurring in the open sea (stable higher salinity) are osmoconformers. The same studies found that the match between the habitat and the OC is consistent along the full life cycle: While marine species are consistently osmoconformers or weak osmoregulators at all life phases, the patterns of osmoregulation vary between larval and juvenile–adult phases depending on habitat. In synthesis, thermally driven changes in osmoregulation and the ontogeny of osmoregulation appear to have evolved in order to match the conditions experienced at each particular life phase or developmental stage. As stated by Weber and Spaargaren (1970), the responses of osmoregulation abilities to temperature appear to be adaptive to the prevailing conditions of temperature and salinity.

**FIGURE 4:**
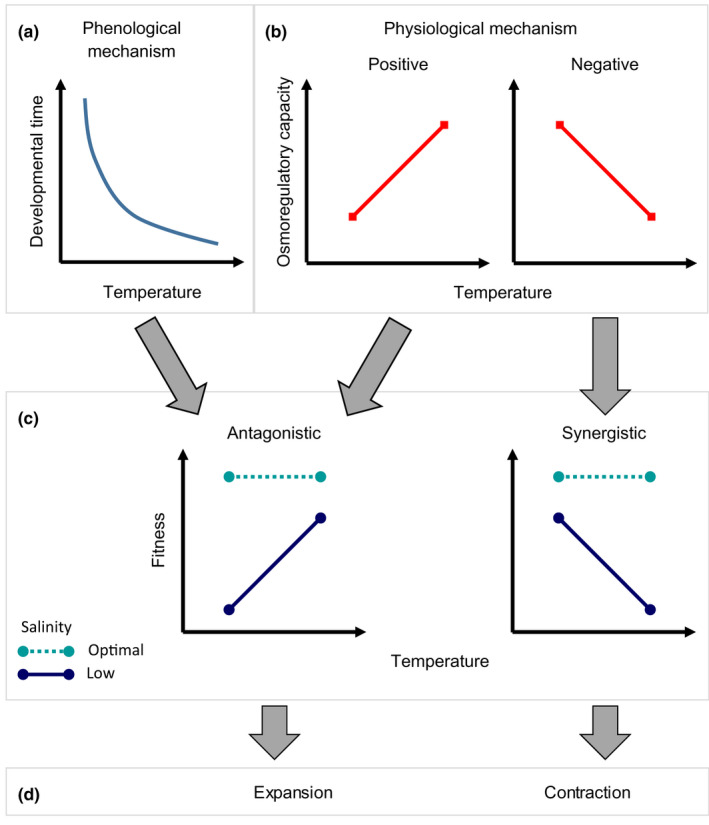
Model describing the contribution of phenological and physiological mechanisms to the responses to effects of temperature and salinity in terms of fitness and species’ distributions. (a) Phenological mechanism: Because increased temperature shortens the developmental time, individuals at high temperatures are exposed to low salinity for a shorter period; in consequence, survival‐at‐stage is higher and the overall response in (c) is antagonistic. (b) Physiological mechanism: When the osmoregulatory capacity (OC) increases with temperature, the fitness response to temperature and salinity in (c) should be antagonistic. When osmoregulatory capacity decreases with temperature, the fitness response in (c) should be synergistic and negative. (d) Contribution of fitness responses to changes in distribution of in coastal‐estuarine species: If temperature increases (and salinity remains constant), an antagonistic pattern should contribute toward expansion toward estuarine waters while the synergistic and negative responses should contribute toward range contraction. Both low salinities and increased temperatures are assumed to be moderate (slightly suboptimal), so as not to become dominant responses; otherwise, the trivial prediction is a dominating effect of either high temperature or low salinity, whichever is stronger

Hence, given the above comparative evidence, we propose a hypothetical model showing the contribution of osmoregulation to responses to salinity and temperature in terms of fitness and species' distributions of coastal marine species of temperate ROFIs (Figure 4); our model also recognizes the contribution of the phenological mechanism. Our predictions are that: (a) In species where elevated temperatures have a positive effect on osmoregulation (e.g., *C. maenas*), osmoregulation should contribute toward TMLS and range expansion toward brackish water habitats (e.g., ROFIs or estuaries) in a warming scenario. (b) The phenological mechanism should also promote TMLS and range expansion toward brackish water habitats in a warming scenario. (c) In species where elevated temperature results in decreased OC, osmoregulation should promote a synergistic negative effect of increased temperature and reduced salinity on fitness (abbreviates a TELS for “thermal exacerbation of low salinity stress”). In those species, and in a warming scenario, reduced osmoregulatory capacity should contribute toward range contraction (i.e., away from brackish water habitats, unless the phenological mechanism prevails). All those predictions are valid only in case of moderate warming, as extreme temperatures would result in a dominating effect of thermal stress in the responses.

### 4.4 Perspectives for model development

The proposed model needs to be tested and expanded, but it can be used as a framework to develop a more general model, considering factors other than osmoregulation. A possible expansion may consider the importance of intracellular regulation (Freire et al., 2008) and permeability to salts; the latter may change with temperature and impact performance, through effects on the amount of energy needed to invest in osmoregulation (Spaargaren, 1975). An additional contributor is required to explain responses where the limiting factor is given at high temperatures: For instance, McLusky (1979) did not find evidence of temperature driving osmoregulation in mysids; yet those adapted to winter conditions had decreased survival in combination of low salinity and high temperatures. For those cases (not covered in our model nor in Figure 4), low salinity and high temperature may impose limitations other than those associated with the uptake of osmotically active substances. The effect of OC on performance may be nonlinear. For instance, in strong osmoregulators (e.g., amphipods) variations in the OC in response to temperature do not always correlate with survival in the laboratory (Dorgelo, 1977), although such drops may impair performance in the field. Care should be taken into considering the “phenological mechanism” yet to be quantified.

In addition, by proposing the above‐referred model, we do not intend to ignore the effects of additional factors on range contraction and expansion (Siren & Morelli, 2020); hence, we must emphasize that our model refers to contributions of osmoregulation and the proposed phenological mechanism. For instance, hypoxia and ocean acidification in coastal areas may become an important factor for performance of marine organisms and it may result from the combined effect of warming and eutrophication (Glober & Baumann, 2016; Vaquer‐Sunyer & Duarte, 2008, 2011). Oxygen limitation for instance may result in changes in the “Pejus temperature,” that is, critical thermal thresholds set by limitations in the capacity of circulatory and respiratory systems to provide oxygen to tissues (Pörtner, 2010). Limitations in oxygen availability are likely to reduce the energy available for osmoregulation, resulting in inhibition of enzymes responsible for ion transport (Lucu & Ziegler, 2017). In consequence, under combined effects of low salinity at high temperatures, oxygen limitation may range from reductions in the strength of TMLS to the induction of TELS. The effect of ocean acidification and salinity may vary among species; those able to osmoregulate appear to withstand moderate levels of acidification (Whiteley et al., 2018). Biotic interactions such as competition between native and exotic species may drive range contractions in native species (Epifanio, 2013). In the case of dispersive larvae, transport by currents plays a central role in determining recruitment of individuals to a given population (Connolly & Roughgarden, 1999) and in mediating effects of climate change on species distributions (Fuchs et al., 2020; Lo‐Yat et al., 2011). Responses to temperature and salinity are likely to define components of performance that are relevant for, for example, competition and larval transport. For instance, competitive abilities can change according to the temperature where such competition occurs (Tomanek & Helmuth, 2002; Watz et al., 2019); comparative studies should provide insights into how the outcome of competition is modulated by responses to combination of temperature and salinity. In addition, larval transport in many crustaceans depends on circatidal or diel migration behavior, which should be driven by the capacity of larvae to accumulate and use energy for vertical swimming. We know that crustacean larvae respond to temperature and salinity as stimuli (Epifanio & Cohen, 2016); however, swimming speed can be modified through physiological effects of both factors (Landeira et al., 2020; Sorochan & Metaxas, 2017; Yu et al., 2010). Overall, physiological information provided by the model may be integrated for instance in metapopulation models (Fordham et al., 2013; Giménez et al., 2020) to consider processes occurring at the individual and population level.

The proposed model should also incorporate the importance of intraspecific variability in the responses to temperature and salinity, as driven by plasticity and selection (Charmantier et al., 2002; Lee et al., 2012). There is currently very little information about the magnitude of intraspecific variation in responses to temperature and salinity in coastal organisms. In *C. maenas*, two recent studies show that the magnitude of TMLS can vary among larvae originated from different females (Spitzner et al., 2019; Torres et al., 2020). In both *C. maenas* and other estuarine species, the tolerance to salinity is driven by the salinity conditions experienced by embryos (Charmantier et al., 2002; Laughlin & French, 1989). Some of those responses appear to be adaptive: For instance in *N. granulata*, the “embryonic effect” produced by low salinity consists in an increased OC and increased survival of the first larval stage. However, in *C. maenas*, low salinity experienced during embryogenesis results in a pre‐emption of TMLS (Torres et al., 2020). Hence, such type of intraspecific variation suggests that conditions existing in the benthic habitat can also affect the capacity of larvae to use estuarine waters. For *C. maenas*, moderately high temperature experienced during embryogenesis promotes TMLS (Torres et al., 2020); thus, moderate increases in temperature both in the maternal and larval habitats may enable larvae the use of waters characterized by moderately low salinity.

Osmoregulatory responses will be relevant for crustaceans and most probably fish, that is, those groups living in aquatic habitats that have evolved the capacity to osmoregulate (Charmantier et al., 2009; Evans & Claiborne, 2009; McCormick et al., 1997). Research on the role of temperature in driving intracellular osmoregulation would be useful to formulate models for invertebrates that osmoconform at low salinities. Importantly, intraspecific variations in the capacity to osmoregulate in temperate species may also explain why we observe both synergistic and antagonistic responses to temperature in marine species (Crain et al., 2008; Przeslawski et al., 2015), that is, because species differ in the nature of the response of osmoregulation capacities to temperature (positive or negative response).

How osmoregulatory responses may drive survival in the field should depend on the covariation of temperature and salinity in ROFIs. For instance, in temperate ROFIs, spring–summer warming of the coastal zone causes shallow waters characterized by reduced salinity to warm up in summer (Gunderson et al., 2016), but cool‐down in winter. In addition, climate change predictions for coastal areas vary regionally concerning salinity (IPCC, 2013), depending on projections of future freshwater runoff from estuaries. Hence, how organisms respond to salinity and temperature will depend on the region, species’ phenology, and ontogenetic patterns in OC. Usually, larval development takes place in spring–summer while the timing of occurrence of juvenile and adults depends on the species life history and migration patterns. Across a sufficiently wide range of temperatures, one would expect a unimodal survival curve driven by thermal tolerance (Pörtner, 2010), with location and spread varying depending on salinity. For instance, on the latitudinal scale, the distribution of *C. maenas* is driven by temperature (Compton et al., 2010), but the question here concerns the use of coastal waters of low salinity (e.g., estuarine plumes or areas such as the Baltic Sea), which may enhance recruitment at the local scale. In invasive species like *C. maenas* (Hines et al., 2004; deRivera et al., 2007), a moderate increase in temperature should be particularly important for expansion in recently invaded areas, to habitats characterized by low salinities.

## 5 CONCLUSIONS

In synthesis, we have found that increased temperature enhances the capacity to osmoregulate in the osmoregulatory larval stages of the shore crab *C. maenas*; correlations between OC and survival suggest that low temperature may constraint the capacity of osmoregulatory mechanisms to work properly and sustain hemolymph osmolality at appropriate levels. The increase in OC works along the phenological effect (increased temperature reduces the time of exposure to low salinity) in mitigating the effects of low salinity on survival.

In addition, we propose a model, for coastal‐estuarine organisms, showing the contribution of osmoregulation and phenological mechanisms into fitness responses to temperature and salinity, as well as responses to warming. OC is a promising integrative measure for understanding the diversity of responses to temperature and salinity and for the prediction of responses of coastal‐estuarine organisms to warming. Tests and expansion of the model are needed to orient research toward the prediction of effects of climate‐driven changes in temperature and salinity on species’ distributions. Given the current status regarding climate change, the “plea for the study of temperature influence on osmotic regulation” done by Verwey (1957) is still valid after almost a century of research in osmoregulation, as it is the plea for the study of the role of temperature on performance at suboptimal salinities.

## CONFLICT OF INTEREST

The authors declare that they have no conflicts of interests. The funding body did not influence the design of the study, the collection, analysis, and interpretation of data nor the writing of the manuscript.

## AUTHOR CONTRIBUTIONS

**Gabriela Torres:** Conceptualization (lead); Data curation (lead); Formal analysis (equal); Funding acquisition (supporting); Investigation (lead); Methodology (equal); Resources (equal); Supervision (lead); Visualization (lead); Writing‐original draft (lead); Writing‐review & editing (equal). **Guy Charmantier:** Conceptualization (equal); Formal analysis (equal); Investigation (equal); Methodology (equal); Writing‐original draft (equal); Writing‐review & editing (equal). **David Wilcockson:** Formal analysis (supporting); Investigation (equal); Methodology (supporting); Writing‐review & editing (supporting). **Steffen Harzsch:** Conceptualization (supporting); Funding acquisition (supporting); Resources (supporting); Writing‐review & editing (supporting). **Luis Giménez:** Conceptualization (equal); Formal analysis (equal); Investigation (supporting); Methodology (supporting); Writing‐original draft (equal); Writing‐review & editing (equal).

## ETHICAL APPROVAL

The research presented in this paper complies with the guidelines from the directives 2010/63/EU of the European parliament and of the Council of 22 September 2010 on the protection of animals used for scientific purposes.

## Supporting information

Table S1‐S8

## Data Availability

The datasets used and/or analyzed during the current study are available from the Dryad Digital Repository (https://doi.org/10.5061/dryad.4tmpg4f97).
