# Supplementary material for: Physiological basis of interactive responses to temperature and salinity in coastal marine invertebrate: Implications for responses to warming
Source: Ecol Evol. 2021 May 1;11(11):7042–56. doi: 10.1002/ece3.7552 (PMC8207410; doi:10.1002/ece3.7552)
Supplement: Supplementary file 1 — Table S1‐S8 [file ECE3-11-7042-s001.docx]

**SUPPLEMENTARY MATERIAL**

**Table S1.** *Carcinus maenas*. Experimental set up. During experiments 1 and 2, larvae (zoeae I or megalopae) were exposed to the experimental temperatures, and acclimation and test salinities until measurement of the osmoregulatory capacity. During experiment 3, zoeae I were exposed to the experimental temperatures and acclimation salinities until sampling for determination of mRNA expression.

| **EXPERIMENT 1: osmoregulation of zoeae I** | | | | | | | | | | | | | | | | |
| --- | --- | --- | --- | --- | --- | --- | --- | --- | --- | --- | --- | --- | --- | --- | --- | --- |
| **Temperature (°C)** | 15.0±0.5 | | | | 18.0±0.5 | | | | 21.0±0.5 | | | | 24.0±0.5 | | | |
| **Acclimation salinities (‰)** | 25.0±0.1 | | 32.5±0.1 | | 25.0±0.1 | | 32.5±0.1 | | 25.0±0.1 | | 32.5±0.1 | | 25.0±0.1 | | 32.5±0.1 | |
| **Test salinities (‰)** | 15.0±0.1 | 20.0±0.1 | 15.0±0.1 | 20.0±0.1 | 15.0±0.1 | 20.0±0.1 | 15.0±0.1 | 20.0±0.1 | 15.0±0.1 | 20.0±0.1 | 15.0±0.1 | 20.0±0.1 | 15.0±0.1 | 20.0±0.1 | 15.0±0.1 | 20.0±0.1 |
|  | | | | | | | | | | | | | | | | |
| **EXPERIMENT 2: osmoregulation of megalopae** | | | | | | | | | | | | | | | | |
| **Temperature (°C)** | 15.0±0.5 | | | | 18.0±0.5 | | | | 21.0±0.5 | | | | 24.0±0.5 | | | |
| **Acclimation salinities (‰)** | 25.0±0.1 | | 32.5±0.1 | | 25.0±0.1 | | 32.5±0.1 | | 25.0±0.1 | | 32.5±0.1 | | 25.0±0.1 | | 32.5±0.1 | |
| **Test salinities (‰)** | 20.0±0.1 | | 20.0±0.1 | | 20.0±0.1 | | 20.0±0.1 | | 20.0±0.1 | | 20.0±0.1 | | 20.0±0.1 | | 20.0±0.1 | |
|  | | | | | | | | | | | | | | | | |
| **EXPERIMENT 3: Expression of genes related to osmotic stress in zoeae I** | | | | | | | | | | | | | | | | |
| **Temperature (°C)** | 15.0±0.5 | | | | 18.0±0.5 | | | | 21.0±0.5 | | | | 24.0±0.5 | | | |
| **Acclimation salinities (‰)** | 25.0±0.1 | | 32.5±0.1 | | 25.0±0.1 | | 32.5±0.1 | | 25.0±0.1 | | 32.5±0.1 | | 25.0±0.1 | | 32.5±0.1 | |

Note: Acclimation and test salinities were prepared by diluting natural seawater (32.5±0.1‰ – 965±1 mOsm*kg^-1^) with appropriate amounts of desalinated water. Salinity was expressed as osmotic pressure (in mOsm kg^-1^) and as salt content of the medium (in ‰); a value of 3.4‰ is equivalent to 100 mOsm kg^–1^ (29.4 mOsm kg^-1^≈1‰) as in Cieluch et al. 2004.

**Table S2.** *Carcinus maenas*. Details of acclimation and test salinities for determinations of osmoregulatory capacity (see main text for details). Values are shown as mean values (n=6) ± standard errors.

| **Acclimation salinities** | | **Test salinities** | |
| --- | --- | --- | --- |
| **‰** | **mOsm kg^-1^** | **‰** | **mOsm kg^-1^** |
| 25.0±0.1 | 729.3±0.9 | 15.0±0.1 | 433.0±0.6 |
| 32.5±0.1 | 965.8±0.9 | 20.0±0.1 | 582.3±0.9 |

**Table S3.** *Carcinus maenas*. Experiment 1: time (in days) of exposure to the acclimation salinities until zoeae I were transferred to the test salinities for 24h (time of exposure started at 50% of the zoea I stage duration). Larvae were kept in the test temperatures (15, 18, 21 and 24°C) and salinities (15 and 20‰) for the 24h prior to the determination of the osmoregulatory capacity. Information is given discriminated by temperature and acclimation salinity. Developmental time to zoea II values were obtained in Experiment 3; shown as mean values for all females (n=4) ± standard errors.

| **Time (days)** | | | | | |
| --- | --- | --- | --- | --- | --- |
|  | **Salinity (‰)** | **Temperature (°C)** | | | |
|  |  | **15.0** | **18.0** | **21.0** | **24.0** |
| **Time until exposure to test salinities** | **25.0** | 4.5 | 3.5 | 2.5 | 2.0 |
|  | **32.5** | 4.0 | 3.0 | 2.5 | 2.0 |
| **Development to zoea II** | **25.0** | 8.9±0.6 | 7.5±0.8 | 5.2±0.4 | 4.9±0.5 |
|  | **32.5** | 7.2±0.2 | 5.2±0.1 | 4.7±0.2 | 4.2±0.1 |

Note: temperature and salinity were adjusted daily to the values given above.

**Table S4.** *Carcinus maenas*. Number of megalopa collected from the field exposed to the acclimation temperatures and salinities for 3 days, before exposure to the test salinity (20‰) for 24h to determine the osmoregulatory capacity.

| **Temperature** | **Acclimation salinity** | |
| --- | --- | --- |
|  | **25‰** | **32‰** |
| 15.0°C | 6 | 6 |
| 18.0°C | 5 | 8 |
| 21.0°C | 5 | 7 |
| 24.0°C | 7 | 6 |

Note: temperature and salinity were adjusted daily to the values given above.

**Figure S1.** *Carcinus maenas*. Variations in the haemolymph osmolality in Zoea I in relation to the osmolality of the medium. Haemolymph osmolality was measured at intermoult (ca. 50% of the moult cycle of the zoea I occurred in the acclimation conditions) after acclimation to 25ppt (blue symbols) and 32.5ppt (green symbols) at 15, 18, 21 and 24°C Values are shown as average values ± standard error (n = 10). Note that standard errors may be smaller than the symbols.

**Table S5.** *Carcinus maenas*. Primers and probes used for gene expression determinations. Genes identified in italics.

|  | **Primer name** | **Primer sequence** |
| --- | --- | --- |
| **Ubiquitin conjugating enzyme E2 L3** |  |  |
| *CamaUB*: Ubiquitin forward | UB-F | TCACCTGGCAGGGACTCATT |
| *CamaUB*: Ubiquitin reverse | UB-R | CCTGAACGCTCCCTTGTTGT |
| *CamaUB*: Ubiquitin probe | UB-P (labelled FAM) | ***FAM***- ACCCGAGAACCCACC |
| **Elongation factor 1-alpha (EF1a)** |  |  |
| *CamaEL*: Elongation factor forward | EL-F | GAGCGGCAGCTATGAGTTCAT |
| *CamaEL*: Elongation factor reverse | EL-R | TGGATGGAGGCTCAATGTTG |
| *CamaEL*: Elongation factor probe | EL-P (labelled VIC) | ***VIC*-**CTCTCTTTGACGCTCTGG |
| **Na^+^-K^+^-ATPase** |  |  |
| *CamaNaK*: Na^+^-K^+^-ATPase forward | NaK-F | CCTCAACCATCTGCTCTGATAAGA |
| *CamaNaK*: Na^+^-K^+^-ATPase reverse | NaK-R | CCACATGTGAGCGACAGTCAT |
| *CamaNaK*: Na^+^-K^+^-ATPase probe | NaK-P (labelled FAM) | ***FAM***-CTCACCCAAAATC |
| **Na^+^-K^+^-_2_Cl^-^-symporter** |  |  |
| *CamaCOT*: Na^+^-K^+^-_2_Cl^-^-symporter forward | COT-F | TCCTGCGTGTGCCTCAAG |
| *CamaCOT*: Na^+^-K^+^-_2_Cl^-^-symporter reverse | COT-R | GGGACAGTCCTCGTCCTCAA |
| *CamaCOT*: Na^+^-K^+^-_2_Cl^-^-symporter probe | COT-P (labelled VIC) | ***VIC-*** CCTGGACTACTCCC |

**Table S6.** *Carcinus maenas*. Amplification efficiency for the primer pairs and probes used for gene expression determinations.

|  | **Slope** | **Amplification factor** | **Amplification efficiency** |
| --- | --- | --- | --- |
| Ubiquitin conjugating enzyme E2 L3 | -3,196 | 2.06 | 105.54 |
| Elongation factor 1-alpha (EF1a) | -3,291 | 2.01 | 101.31 |
| Na^+^-K^+^-ATPase | -3,319 | 2.00 | 100.12 |
| Na^+^-K^+^-_2_Cl^-^-symporter | -3,397 | 1.97 | 96.96 |

Note: Slopes to calculate the amplification factors and efficiencies were obtained using GraphPad software from standard curves of serial diluted cDNA from adult gills. Amplification factors and efficiencies were calculated using the Amplification efficiency calculator Thermofisher Scientific:

<https://www.thermofisher.com/de/de/home/brands/thermo-scientific/molecular-biology/molecular-biology-learning-center/molecular-biology-resource-library/thermo-scientific-web-tools/qpcr-efficiency-calculator.html?gclid=EAIaIQobChMIzPvqoeSw6QIVBc93Ch2C-QivEAAYASAAEgKTWvD_BwE&ef_id=EAIaIQobChMIzPvqoeSw6QIVBc93Ch2C-QivEAAYASAAEgKTWvD_BwE:G:s&s_kwcid=AL!3652!3!394297685910!b!!g!!?cid=bid_mol_eps_r01_co_cp1358_pjt0000_bid00000_0se_gaw_dy_pur_con>

**Table S7.** *Carcinus maenas*. Model selection for relative expression of the osmotic stress-related genes: enzyme Na^+^-K^+^-ATPase (NaK) and protein Na^+^-K^+^-_2_Cl^2-^-symporter (COT) for zoeae I). Model selection was carried out using the adjusted Akaike information criteria (AICc). Selection for random terms was based on restricted maximum likelihood fitting (REML) while that of fixed terms was based on maximum likelihood (ML) fitting. Abbreviations: ♀: female of origin; T: temperature; S_A_: acclimation salinity. The best overall model contains both the best random and fixed term as highlighted in bold: for example, the best model for NaK and COT contained female of origin as a random term (i.e. random intercepts) and acclimation salinity or temperature as a fixed term for NaK or COT, respectively.

| Model selection: Random (REML) | AICc | |
| --- | --- | --- |
| Term | NaK | COT |
| ♀:T:S_A_ | 104 | 195 |
| ♀:T | 87 | 184 |
| ♀:S_A_ | 92 | 190 |
| ♀ | **84** | **183** |
| Model selection: Fixed (ML) | | |
| T:S_A_ | 62 | 169 |
| T+S_A_ | 56 | 163 |
| T | 59 | **163** |
| S_A_ | **55** | 171 |
| Null | 58 | 171 |

**Figure S2.** *Carcinus maenas*. Effects of acclimation salinity and temperature (Experiment 3) on relative expression of mRNA of Na^+^-K^+^-ATP (left panel) and Na^+^-K^+^-_2_Cl^-^ symporter (right panel) in zoeae I. Data are shown as average values ± SE for all four females (thick lines), and discriminated by each individual female (thin lines). Values corresponding to acclimation to 25.0‰ are shown in blue (average: full thick line and light blue squares; discriminated by female: full thin line and crosses). Values corresponding to acclimation to natural seawater (32.5‰) are shown in green (average: dashed thick line and light green circles; discriminated by female: dashed thin line and dark circles).

**Table S8.** *Carcinus maenas*. Instantaneous mortality rates (µ in days^-1^) and comparisons of patterns of responses to the combination of salinity and temperature in survival to the second zoeal stage for larvae hatched from 10 different females. Re-analysis of data from Figure 3 of Spitzner et al. (2019). Focus is given to survival patterns at 20 ppt, which are those showing TMLS. Columns: **TMLS**: whether survival patterns consistent with thermal mitigation of low salinity stress (see Fig. 3 in Spitzner et al. 2019); **peak survival**, temperature at which survival peaks in larvae exposed to 20 ppt (*: high survival over the full range of salinities tested); **µ (15 °C)**: instantaneous mortality rates calculated for the lowest temperature (15°C); **μ (21 °C)**: instantaneous mortality rates calculated for 21°C. Cases where TMLS were observed are highlighted in bold: for those cases, instantaneous mortality rates were lower at the highest temperature, showing that the effect of temperature on developmental time alone cannot explain the increased survival to zoea II.

| Female | TMLS | Peak survival | μ(15°C) x 10^-2^ | μ(21°C) x 10^-2^ |
| --- | --- | --- | --- | --- |
| 1 | **Yes** | **21** | **8.77** | **1.39** |
| 2 | **Yes** | **21** | **22.55** | **10.75** |
| 3 | No | 15-24* | 2.54 | 3.65 |
| 4 | No | 18 | 11.61 | 11.60 |
| 5 | **Yes** | **21** | **7.81** | **2.21** |
| 6 | No | 15 | 2.25 | 4.73 |
| 7 | No | 18 | 4.88 | 8.02 |
| 8 | **Yes** | **21** | **13.37** | **7.23** |
| 9 | No |  | 13.81 | 20.30 |
| 10 | **Yes** | **21** | **16.70** | **6.85** |
